# What's the meaning of local? Using molecular markers to define seed transfer zones for ecological restoration in Norway

**DOI:** 10.1111/eva.12378

**Published:** 2016-04-06

**Authors:** Marte Holten Jørgensen, Abdelhameed Elameen, Nadine Hofman, Sonja Klemsdal, Sandra Malaval, Siri Fjellheim

**Affiliations:** ^1^ Department of Plant SciencesNorwegian University of Life SciencesÅsNorway; ^2^ Norwegian Institute of Bioeconomy ResearchÅsNorway; ^3^ Conservatoire Botanique National des Pyrénées et de Midi‐PyrénéesBagnères‐de‐BigorreFrance

**Keywords:** ecological restoration, gene flow, genetic diversity, local seeds, seed transfer zones, site‐specific seeds

## Abstract

According to the Norwegian Diversity Act, practitioners of restoration in Norway are instructed to use seed mixtures of local provenance. However, there are no guidelines for how local seed should be selected. In this study, we use genetic variation in a set of alpine species (*Agrostis mertensii, Avenella flexuosa, Carex bigelowii, Festuca ovina, Poa alpina* and *Scorzoneroides autumnalis*) to define seed transfer zones to reduce confusion about the definition of ‘local seeds’. The species selected for the study are common in all parts of Norway and suitable for commercial seed production. The sampling covered the entire alpine region (7–20 populations per species, 3–15 individuals per population). We characterised genetic diversity using amplified fragment length polymorphisms. We identified different spatial genetic diversity structures in the species, most likely related to differences in reproductive strategies, phylogeographic factors and geographic distribution. Based on results from all species, we suggest four general seed transfer zones for alpine Norway. This is likely more conservative than needed for all species, given that no species show more than two genetic groups. Even so, the approach is practical as four seed mixtures will serve the need for restoration of vegetation in alpine regions in Norway.

## Introduction

In many cases, natural succession is sufficient to restore an area to its original state after anthropogenic disturbance (e.g. Prach and Pysek 2001). However, in areas where succession is slow and risk of erosion is high, there is a danger of reinvasion of non‐native species or for aesthetical and technical reasons seeding to restore vegetation may be necessary. Seeds of local provenance are widely recommended for restoration projects for reasons that include avoiding genetic contamination of local populations, increasing restoration success through better seedling establishment, survival and growth of locally adapted plant material and to avoid outbreeding depression (reviewed in Broadhurst et al. 2008). There is, however, no general agreement on what local means simply because it will vary with species, goals and technicality of each individual restoration project (Linhart and Grant 1996; McKay et al. 2005; Perring et al. 2015).

Ecosystems at high latitudes and altitudes are especially vulnerable to human interference. Due to short growing seasons, low temperatures and often dry and nutrient‐poor soils, the natural process of revegetation may take decades (Krautzer et al. 2012). Consequently, erosion may often exceed damaging effects of the initial anthropogenic disturbances (Vasil'evskaya et al. 2006). Several assessments of revegetation indicate that the vegetation cover needs to exceed 70–80% to reduce soil erosion to an acceptable degree in these habitats (Markart et al. 1997; Tasser et al. 1999; Peratoner 2003), and establishment of such a vegetation cover within reasonable time is crucial. Because natural revegetation processes are so slow, human intervention is necessary to avoid erosion (e.g. Krautzer et al. 2012). In Norway, approximately 30% of the mainland is above or north of the climatic forest line (www.biodiversity.no); thus restoration of vegetation by seeding is often necessary.

**Figure 1:**
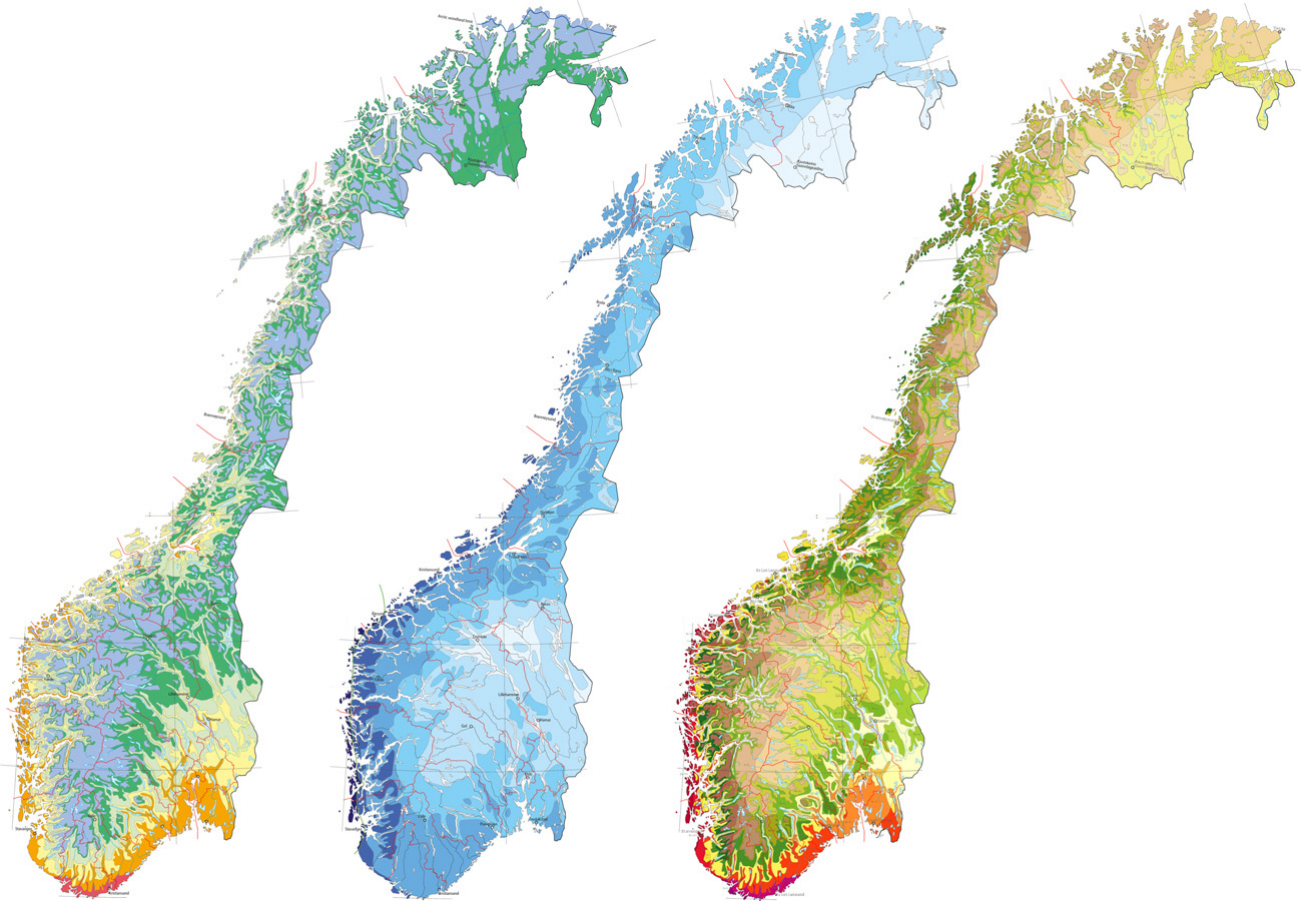
Vegetation zones (left), sections (middle) and zone sections (right) in Norway, reflecting our main gradients, the latitudinal and altitudinal gradients (left), the oceanity gradient (middle) and the combination of these (right). The zones are the nemoral (red), the boreo‐nemoral (orange), the boreal (yellow, bright green, green) and the alpine (blue). The sections are categorised from highly oceanic (dark blue) to mildly continental (white). The figure is taken from Moen (1998) with a few modifications by Halvorsen et al. (2009).

The Norwegian flora is shaped by three main gradients: the latitudinal, the altitudinal and the oceanity gradients. In combination with the complex topography, these gradients create vegetation zones which are mosaic‐like in distribution (Fig. 1). The flora is relatively young, as the area was covered by the Weichselian ice sheath until 11 k years ago (Påsse and Andersson 2005). The flora has low biodiversity with only 3000 species (Elven 2005) and contains few endemisms (Borgen 1987). Most species are in the outskirts of their distribution ranges (Hultén and Fries 1986). Studies of phylogeography of Norwegian species suggest little or no genetic structure in neutral markers, reflecting the young history and isolation of the Norwegian flora (Schönswetter et al. 2003, 2008; Fjellheim and Rognli 2005; Alsos et al. 2007; Gaudeul et al. 2007; Elameen et al. 2008b; Vik et al. 2010; Westergaard et al. 2010, 2011; Bjørgaas 2011).

Restoration projects in Norway must follow the legal framework set by the Norwegian Nature Diversity Act of 2009 (https://lovdata.no/dokument/NL/lov/2009-06-19-100?q=naturmangfoldloven. Associated regulations from 2015: https://lovdata.no/dokument/SF/forskrift/2015-06-19-716). The aim of the law is to preserve nature as it is, even down to maintaining genetic integrity on a population level. Following this, there is a legal demand for material of local provenance. However, there are no guidelines for what local means, and practitioners and users are asking for clarifications.

### Different strategies for the definition of seed transfer zones

To approach the demand for local seeds, we may restrict plant translocation to seed transfer zones within which plant materials can be moved freely with minimal loss of biodiversity and local adaptation (Knapp and Rice 1994; Jones 2003; McKay et al. 2005; Vander Mijnsbrugge et al. 2010; Miller et al. 2011). Many authors have proposed methods to define them for different kinds of species and at different scales, resulting in several distinctive delineation strategies (Mahalovich and McArthur 2004; McKay et al. 2005; Vander Mijnsbrugge et al. 2005). The different strategies are not mutually exclusive and may well be combined to cover several aspects of revegetation.

One of the strategies is the ecoregional approach. Drawn on topographic, climatic or edaphic data for zones of ecological similarity, the zones encompass geographic areas with similar ecological conditions, such as geology, climate, vegetation, soils and hydrogeology (Mahalovich and McArthur 2004). Ecoregional seed transfer zones were first defined in recognition of strong regional differences in life‐history traits for commercially important tree species (Millar and Libby 1989; Hufford and Mazer 2003; Vander Mijnsbrugge et al. 2005; Miller et al. 2011). To apply the ecoregional approach of seed zone definition in the complex landscape of Norway (Fig. 1) would be both difficult and impractical.

Another strategy is to use an adaptive focus. To ensure the technical success of restoration, the best adapted plant population for the target area is often used as seed source (Bischoff et al. 2006; Leimu and Fischer 2008; Rice and Knapp 2008; Wilson et al. 2008; Hereford 2009). To quantify adaptive potential of the populations seeds of different origin are tested in common garden experiments (Kitchen and McArthur 2001; Johnson et al. 2004; Kawecki and Ebert 2004; Miller et al. 2011). Such adaptive effect differentiation is documented in some plant populations (Sahli et al. 2008; Bischoff et al. 2010); however, there are also examples of the opposite (e.g. Fjellheim et al. 2015). The largest challenge in alpine regions in Norway is seedling establishment and rapid creation of vegetation cover in a harsh environment prone to erosion. Using adapted seed material may be of paramount importance for restoration in alpine areas of Norway, but may not necessarily preserve genetic integrity of local populations as it has been shown that in some cases, the best adapted populations are not local (Bischoff et al. 2010; Jones 2013).

A third approach that may best fulfil the intention of the Nature Diversity Act to preserve genetic integrity of local flora is to use gene flow patterns for seed zone design. It involves a goal of maintaining the natural spatial genetic structure of the species, as well as preserving genetic diversity to ensure long‐term population survival and reproduction (McKay et al. 2005). The history of a population and the landscape within which it exists are critical factors influencing the genetic relationships of populations (Krauss and Koch 2004). Genetic structure results from the joint action of mutation, migration, inbreeding, selection and drift, which in turn must operate within the historical and biological context of each plant species (Loveless and Hamrick 1984). Neutral markers have commonly been used to reflect gene flow and genetic drift, and have been useful for defining seed transfer zones for the conservation of continuous plant populations (Moritz 1999; Diniz‐Filho and Telles 2002; Krauss and Koch 2004; Malaval et al. 2010). However, neutral markers do not normally reflect adaptive variation (Holderegger et al. 2006), and additional studies such as common garden studies of potentially important traits or genome‐wide scans to detect adaptation to climate (Steane et al. 2014) are needed to identify locally adapted plant populations.

The science and practice of ecological restoration have raised high expectations for our ability to reverse the loss of biodiversity and ecosystem services (Mijangos et al. 2014). Realistically, decision‐making in restoration is based on incomplete knowledge (Rice and Emery 2003), and our governments are still in need of practical and efficient tools for management and preservation. Genetic tools from conservation genetics and related research areas can improve the practice of ecological restoration by providing data on population expansions and contractions, historical gene flow and coalescence (Mijangos et al. 2014). An understanding of the various processes involved in shaping the genetic structure of a population will increase the short‐ and long‐term success of conservation and restoration efforts (Rice and Emery 2003).

The main aim of this study was to provide a scientific basis for selection of local seeds for restoration of vegetation in alpine regions in Norway in compliance with the Norwegian Nature Diversity Act. To circumvent the need for time‐ and cost‐consuming reciprocal transplant and common garden trials to identify well‐adapted seed material, but still ensure good seedling establishment, we chose to work with a set of common species already in commercial seed production and regularly used in restoration projects, but as of today not necessarily in compliance with the Norwegian Nature Diversity Act. We used molecular markers and population genetic tools to identify genetic groups for the species and compare the groups to suggest general seed transfer zones that match the genetic structures found in all species. The resulting generalised seed transfer zones provide a basis for selection of local seeds for most alpine vegetation reconstructions in Norway in foreseeable future.

## Materials and methods

### Collection of plant materials

**Figure 2:**
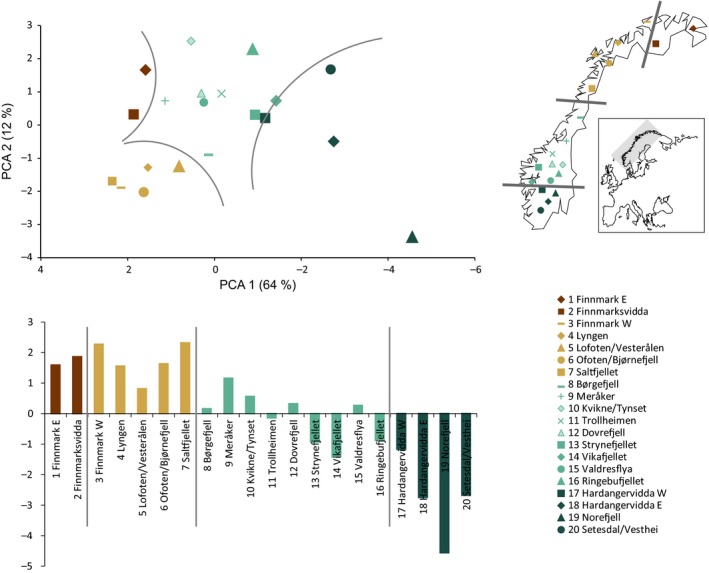
Sampling localities included in this study (to the right), and cluster analysis (to the left) of all localities based on a principal component analysis of the mean PCO scores for all populations and all species included in this study. Above: Scatterplot of the first two axes, PCA 1 (64%) and PCA 2 (12%). Below: PCA 1 scores for all localities sorted by geography.

Plant material (leaves) was collected in natural habitats from 20 locations throughout Norway in 2009 and 2011 (Fig. 2; Tables 1 and S1). The collection and the choice of the model species were published in Jørgensen et al. (2014) and were based on the following criteria: (i) plant materials must be fresh and disease‐free, (ii) growing distance between individual plants within collection sites must be at least 5–10 m, (iii) collection of the species should not take place in an area where previous seeding or introduction of the species may have occurred as result of re‐vegetation, (iv) high growth rate (ensures quick establishment of vegetation cover) (v) a minimum of 20 individual plants of each species per location and (vi) the species are already in use in commercial seed production (ensures good seed production). The six species chosen for the study are *Agrostis mertensii* Trin., *Avenella flexuosa* (L.) Parl., *Carex bigelowii* Torrey ex Schweinitz, *Festuca ovina* L., *Poa alpina* L. and *Scorzoneroides autumnalis* (L.) Moench. A total of 151–300 individuals of each species were sampled throughout Norway (Table 1). After collection, the plant materials were stored in individual zip‐lock bags containing silica gel.

**Table 1:** Sampling for each species included in this study, individuals per population. Lat./Long. give approximate coordinates for each locality, north and east. See Table S1 for further details

Locality	Lat./Long. (N/E)	*Agrostis mertensii*	*Avenella flexuosa*	*Carex bigelowii*	*Festuca ovina*	*Poa alpina*	*Scorzoneroides autumnalis*
1) Finnmark E	70.27/30.96	15	15	15	15	7	–
2) Finnmarksvidda	69.40/24.53	14	15	14	–	–	–
3) Finnmark W	71.08/25.75	–	15	15	14	11	15
4) Lyngen	69.60/20.24	–	15	14	–	4	15
5) Lofoten/Vesterålen	68.34/14.65	15	15	–	–	7	15
6) Ofoten/Bjørnefjell	68.45/18.10	15	15	11	14	13	15
7) Saltfjellet	67.07/16.05	15	15	12	15	14	5
8) Børgefjell	65.18/13.46	14	15	12	–	–	14
9) Meråker	63.36/11.74	–	15	14	14	9	15
10) Kvikne/Tynset	62.57/10.45	–	15	15	14	6	15
11) Trollheimen	62.71/9.55	–	15	14	15	13	13
12) Dovrefjell	62.30/9.60	–	15	14	13	15	15
13) Strynefjellet	62.02/7.40	15	15	14	–	–	14
14) Vikafjellet	60.93/6.43	15	15	15	–	13	11
15) Valdresflya	61.34/8.81	15	15	10	14	–	15
16) Ringebufjellet	61.58/10.36	–	15	15	15	10	15
17) Hardangervidda W	60.43/7.41	15	15	–	14	–	15
18) Hardangervidda E	60.24/8.53	15	15	14	14	–	15
19) Norefjell	60.34/9.19	15	15	13	14	15	15
20) Setesdal/Vesthei	59.46/7.19	13	15	8	–	14	3
Total no. of specimens		191	300	239	185	151	240

### DNA extraction

Silica gel‐dried leaf tissue and one 3‐mm Tungsten Carbide Bead (QIAGEN Inc., Valencia, CA, USA), were placed in a 96‐well plate and kept for 3 min in liquid nitrogen. The plates were shaken twice in a Mixer‐mill disruptor MM301 (Retsch, Haan, Germany) for 90 s at 25 Hz. DNA was extracted, using the Plant DNA Kit of Omega Bio‐tek (Norcross, GA, USA) according to the manufacturer's instructions.

### AFLP analysis

The AFLP analysis (Vos et al. 1995) was performed as previously described (Elameen et al. 2008a; Jørgensen et al. 2014), with modifications that included the use of fluorescently labelled primers instead of radioactive labelling. Six amplification primer pairs with two selective bases were tested using 10 individuals for each species. Four of these (Table 2; Applied Biosystems, Foster City, CA, USA and Invitrogen, Carlsbad, USA) were chosen based on the number of amplified fragments in the range 50–500 base pairs, and amount of polymorphism among the included individuals.

**Table 2:** Sequences of the *Eco*RI and *Mse*I selective primers used for AFLP analysis

Primer combination	EcoRI primer 5′‐3′	MseI primer 5′‐3′
EcoRI_0_ × MseI_0_	GACTGCGTACCAATTC	GATGAGTCCTGAGTAA
EcoRI_12_ × MseI_17_	6FAM‐GACTGCGTACCAATTCAC	GATGAGTCCTGAGTAACG
EcoRI_19_ × MseI_17_	6FAM‐GACTGCGTACCAATTCGA	GATGAGTCCTGAGTAACG
EcoRI_20_ × MseI_17_	6FAM‐GACTGCGTACCAATTCGC	GATGAGTCCTGAGTAACG
EcoRI_21 _× MseI_17_	6FAM‐GACTGCGTACCAATTCGG	GATGAGTCCTGAGTAACG

### Data scoring

Data were recorded manually using GeneMapper 5 (Applied Biosystems), and only clear polymorphic bands were scored for presence (1) or absence (0). The results of AFLP were confirmed by repeating the analyses of 23 randomly selected plants of each of the six species. The replicated profiles were compared, and markers with more than 5% errors were removed from the data sets. Also single profiles with significantly higher or lower number of bands compared to the average were removed as we assumed that to be the result of imperfect PCRs.

### Statistical analyses

Our main goal was to define seed transfer zones in Norway for the selected species. To do so, we needed to identify geographic structure and analyse the diversity for each taxon. To identify geographic structure, we used two approaches. First, we visualised the genetic variation using an ordination method, principal coordinate analysis (PCO) as we had binary matrices. The analyses were conducted using the software PAST (Hammer et al. 2001) and Dice's similarity index (Dice 1945). Second, we used a nonhierarchical clustering method that grouped the individuals to maximise linkage disequilibrium among groups, that is we assumed the same pattern in several markers across group barriers, whereas within groups, the patterns should be random. The groups were identified using the Bayesian program Structure v 2.1 (Pritchard et al. 2000; Falush et al. 2003). Plots of likelihoods, similarity coefficients and ΔKs (Evanno et al. 2005) were made in the statistical package R (http://www.r-project.org/) using the script Structure‐sum (Ehrich 2006). To analyse the diversity patterns, we used analysis of molecular variance (amova) in the program Arlequin v 3.11 (Excoffier et al. 1992, 2005) that calculated the variation within and among prior defined populations. We also ran Mantel tests (Mantel 1967) for correlations between genetic and geographic distance matrices in Arlequin.

To visualise patterns among geographical localities, we conducted a meta analysis where mean PCO scores for each population and each species (i.e. mean population values for the first two eigenvectors) were used as input in a principal component analysis (PCA) in PAST.

## Results

### *Agrostis mertensii*

**Figure 3:**
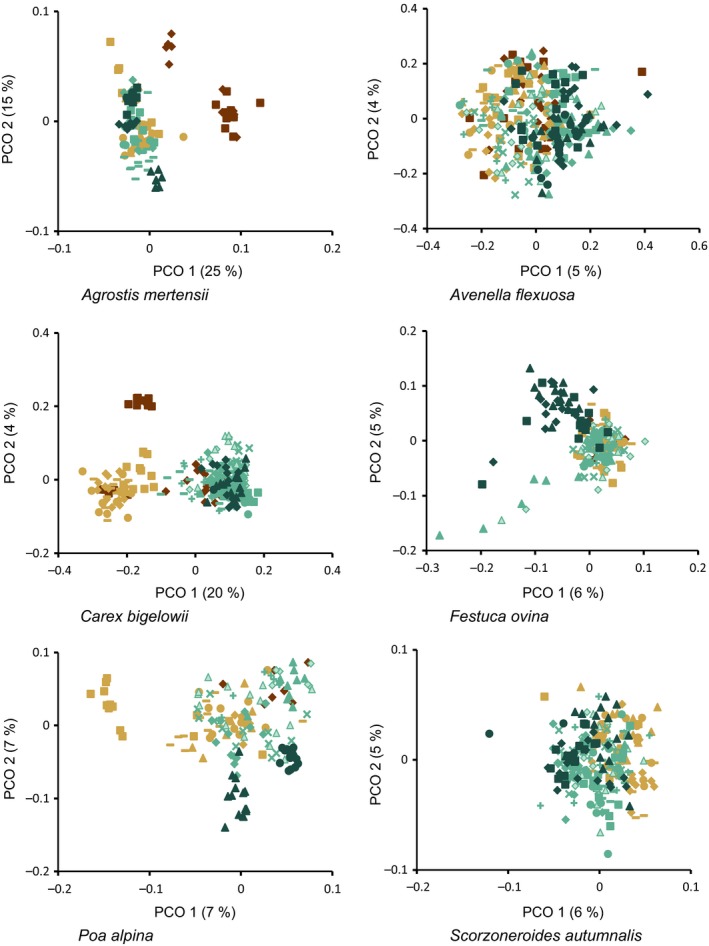
Principal coordinate analyses for all species included in this study. Eigenvalue for each axis is given in brackets. See Fig. 2 for a legend of symbols/colours.

The ordination analysis separated the two northernmost populations (Finnmark E and Finnmarksvidda) from the remaining along the first two axes (25 and 15%, respectively; Fig. 3). No further structure could be identified. In the Structure analyses, the likelihoods, similarities and ΔKs all suggested a clustering into three groups (Fig. S1): one consisting primarily of the northernmost populations (Finnmark E and Finnmarksvidda), the other two overlapping, but with one dominating Central Norway, and the other dominating southern Norway (Fig. S2). The amova analysis showed that 52% of the variation was among populations, whereas 48% was within population variation (Table 3). The Mantel test showed no significant relation between genetic and geographic distance.

**Table 3:** amova analyses for the six species included in this study. Only percentage of variation is shown. All components were significant with *P* < 0.05

Species	Among population variation (%)	Within population variation (%)
*Agrostis mertensii*	52	48
*Avenella flexuosa*	10	90
*Carex bigelowii*	30	70
*Festuca ovina*	11	89
*Poa alpina*	28	72
*Scorzoneroides autumnalis*	12	88

### *Avenella flexuosa*

No apparent groups were identified in the ordination analysis, but a gradient from North to South could be seen along the first two axes (5 and 4%, respectively; Fig. 3). In the Structure analyses, the likelihoods, similarities and ΔKs all suggested a clustering into a single group (Figs S1 and S2). The amova analysis showed that only 10% of the variation was among populations, whereas 90% was within population variation (Table 3). The Mantel test showed no significant relation between genetic and geographic distance.

### *Carex bigelowii*

The populations were grouped into two groups along the first two axes of the PCO (20 and 4%, respectively); a northern group from Saltfjellet northwards, and a southern group from Bjørgefjell southwards (Fig. 3). However, the northeasternmost population from Varanger/Finnmark E grouped with the southern group. In the Structure analyses, the likelihoods, similarities and ΔKs all suggested a clustering into two groups (Fig. S1): one consisting primarily of the populations from Saltfjellet and northwards, the other primarily of the populations from Bjørgefjell and southwards (Fig. S2). The amova analysis showed that 30% of the variation was among populations, whereas 70% was within population variation (Table 3). The Mantel test showed no significant relation between genetic and geographic distance.

### *Festuca ovina*

The ordination analysis separated the southernmost populations (Hardangervidda E and W, and Norefjell) from the remaining along axes one and two (6 and 5%, respectively; Fig. 3). No further structure could be identified. In the Structure analyses, the likelihoods, similarities and ΔKs all suggested a clustering into two groups (Fig. S1): one consisting primarily of the populations from Hardanger (E and W) and Norefjell, and the other of the remaining populations (Fig. S2). The amova analysis showed that 11% of the variation was among populations, whereas 89% was within population variation (Table 3). The Mantel test showed no significant relation between genetic and geographic distance.

### *Poa alpina*

The ordination analysis separated the Saltfjellet population from the remaining along axis one (7%), and partly the southernmost populations (Setesdal/Vesthei and Norefjell) from the remaining along axis two (7%; Fig. 3). In the Structure analyses, the likelihoods, similarities and ΔKs all suggested a clustering into three groups (Fig. S1): one consisting primarily of the Saltfjellet population, one consisting of the two southernmost populations (Setesdal/Vesthei and Norefjell), and the third consisting of the remaining populations (Fig. S2). The amova analysis showed that 28% of the variation was among populations, whereas 72% was within population variation (Table 3). The Mantel test showed no significant relation between genetic and geographic distance.

### *Scorzoneroides autumnalis*

No apparent groups were identified in the ordination analysis, but a gradient from North to South could be seen along the first two axes (6 and 5%, respectively; Fig. 3). In the Structure analyses, the likelihoods, similarities and ΔKs all suggested a clustering into two groups (Fig. S1): one domination in northern Norway, the other in the South, but overlapping (Fig. S2). The amova analysis showed that 12% of the variation was among populations, whereas 88% was within population variation (Table 3). The Mantel test showed no significant relation between genetic and geographic distance.

### The meta analysis

When running a PCA on the mean PCO scores for each population and species, no clear groups of the localities could be identified. However, they did form a gradient along the first PCA axis (64%) with the southernmost populations at the low end and the northernmost populations at the high end (Fig. 2).

## Discussion

### Delineation of species specific seed transfer zones

Four of the six species (*Poa alpina, Festuca ovina, Scorzoneroides autumnalis* and *Avenella flexuosa*) show shallow spatial structuring of genetic variation with the two first axes in the PCO explaining less than 15% of the variation (Fig. 3), and most of the genetic variation in these species is found within populations (Table 3). *Avenella flexuosa* and *S. autumnalis* show no clear structuring of the populations; however, a south–north gradient can be seen in the PCO. The Structure analysis of *S. autumnalis* suggests a division into two genetic groups, one mainly southern and one mainly northern (Figs S1 and S2). Nevertheless, no sign of isolation by distance was detected by Mantel tests and we suggest a single seed zone in Norway for each of these species. *Festuca ovina* and *P. alpina* show weak differentiations of the southernmost populations compared to the northern ones. The transition corresponds with a major change in bedrock and may relate to that (Norwegian Geological Survey 1984). Considered separately, each species would probably have been identified as a single genetic group given the low percentage of variation explained and little differentiation between the populations. However, the congruence of the structuring of variation in the two species supports a separate seed zone south of Hardangervidda. Population 7 of *P. alpina* (from Saltfjellet) is separated from the remaining populations. *Poa alpina* is known to have mixed reproductive strategies, with some populations reproducing apomictically and some sexually (Müntzing 1965). Apomixis would reduce gene exchange with other populations, and may explain the differentiation. Given the overall lack of differentiation, it is unlikely that this population represents a population with a separate history, and we propose not to define the Saltfjellet area as a separate seed transfer zone. As a precautionary measure, *P. alpina* could be excluded from restoration projects and seed source populations in this area.

**Figure 4:**
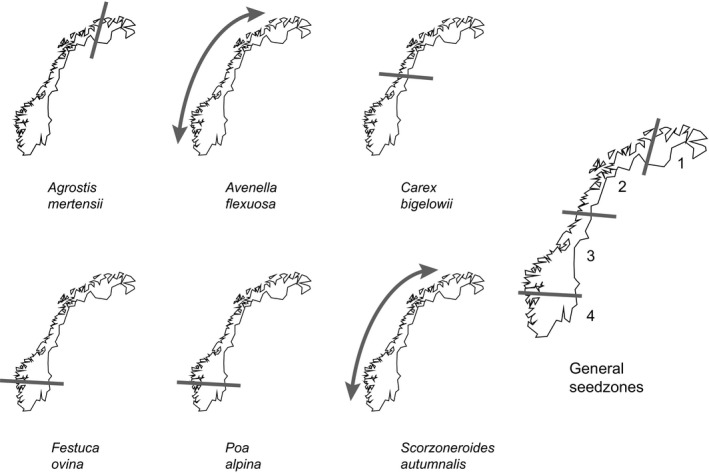
Suggested seed transfer zones for each species included in this study, and suggested overall seed transfer zones.

In contrast to the weak genetic structuring identified in *P. alpina*,* F. ovina*,* A. flexuosa* and *S. autumnalis*, the genetic diversity of *C. bigelowii* is clearly structured into two groups, one northern and one southern (Figs 3 and 4), in accordance with previous findings (Schönswetter et al. 2008). The area where the two groups meet is a well‐known contact area for both plants and animals in the middle of Fennoscandia (e.g. Taberlet et al. 1998; Hewitt 1999; Brochmann et al. 2003; Schmitt 2007) and corresponds to where the icecap of the Weichselian longest prevailed (Påsse and Andersson 2005). The two groups probably represent two of the main immigration routes to Norway after the ice age: an eastern element migrating from Russia and a southern element migrating from Central Europe. *Carex bigelowii* mainly reproduces vegetatively by runners (Callaghan 1976), and this may contribute to reduced gene flow between the two groups, maintaining the structure of genetic diversity. The one population (in Finnmark) that is completely separated from the remaining is probably introduced. Many species were brought to this area from Germany during World War II (polemochores), and *C. bigelowii* may well have been one of them (Alm et al. 2009; Alm personal communication). Therefore, we choose not to let it influence the definition of seed transfer zones.

The populations of *Agrostis mertensii* separate into two distinct geographic groups in the PCO analysis with a border west of the high mountain plateau of Finnmarksvidda (Figs 3 and 4), whereas the Structure analysis further divides the southern group into two (Figs S1 and S2). The large differences between the populations are also reflected in the amova analysis (Table 3). We may explain the differentiation between groups with reproductive strategy or phylogeographic history. We have, however, not been able to find any information about the reproductive biology of *A. mertensii*, so we are unable to confirm this.

Large amount of gene flow may account for the low level of genetic structuring and lack of signal in Mantel tests in *P. alpina*,* F. ovina*,* A. flexuosa* and *S. autumnalis* as they are all wind‐pollinated (*P. alpina*,* F. ovina*,* A. flexuosa*) or wind‐dispersed (*S. autumnalis*). The three species with the least differentiation between the populations (*F. ovina*,* A. flexuosa* and *S. autumnalis*) are distributed also in lowland parts of Norway, and the connectivity between alpine regions most likely facilitates gene flow between the populations. Furthermore, the Norwegian populations of *A. flexuosa*,* S. autumnalis* and *F. ovina* are part of a larger, continuous geographic distribution of the species (Hultén and Fries 1986) covering all of Europe and large parts of Asia ensuring high effective population sizes and probably gene flow to the populations from several directions, working against genetic differentiation of populations as seen in the analyses of molecular variance. Similar results were found in the widespread, wind‐pollinated *Phleum pratense*, where no structuring of genetic variation (SSR) could be found in its entire Eurasian distribution area (Fjellheim et al. 2015). The distribution of *A. mertensii*,* C. bigelowii* and *P. alpina* is restricted to alpine regions in Norway, and the lack of continuous distribution may limit gene flow between the populations and account for the larger between‐population variation. The geographic distribution ranges of *A. mertensii*,* C. bigelowii* and *P. alpina* are limited in comparison with *A. flexuosa*,* S. autumnalis* and *F. ovina,* possibly reducing the influx of genetic material to the populations, and increasing the among population variation.

Our results suggest that four seed transfer zones suffice for all species included in the study (Fig. 4). When combining the results from all species in a meta analysis, the sampling localities are structured according to geographical distance (Fig. 2). The transitions between the zones follow a latitudinal gradient with borders along 61 and 66° north in the southern part of the country and a third line west of the mountainous plateau of Finnmarksvidda. The geographical limits of the four zones are of course approximate, limited by the spatial resolution of the sampling and the gradational nature of the transitions. When considering a single species, the number of zones is larger than warranted; however, the four zones are not in conflict with any of the genetic patterns that we find (Fig. 4). Furthermore, the structure we do find is shallow, reflecting the young age of the Norwegian flora. The division into general seed transfer zones instead of single zones for each species creates a practical tool for environmental management and is possible to implement for seed producers and end users.

Restoration ecologists have put much focus on defining seed transfer zones based on adaptation. To increase the chance of success of establishing vegetation cover, the best adapted population for the restoration area is identified by testing seeds of different origins in common garden experiments to quantify home seed advantages (Kitchen and McArthur 2001; Johnson et al. 2004; Kawecki and Ebert 2004; Bower and Aitken 2008; Miller et al. 2011). However, the scale at which we find local adaptation is highly variable among species and populations and is dependent on distribution, mode of dispersal and reproduction, and evolutionary and life history (Lenssen et al. 2004; Bischoff et al. 2006; Broadhurst et al. 2008; Leimu and Fischer 2008). The species we included in our study are widely distributed, abundant and either wind‐pollinated or wind‐dispersed; thus gene flow is common also on a large scale, and adaptation is probably also large‐scaled. *Phleum pratense,* a common, widely distributed grass species which has similar life‐history strategies as our species (wind‐pollination and wind‐dispersal) shows no sign of local adaptation within the Nordic region (Fjellheim et al. 2015).

The species chosen for the study are known to be easily established and have high growth rate as they have already been used for restoration projects in Norway, however, not in compliance with the Nature Diversity Act as seeds has not necessarily been of local provenance. The proposed system answers the call in the Norwegian Nature Diversity Act for seeds of local provenance. If, for certain areas, specific adaptations are required, we suggest that our seed transfer zones are used as a framework, and that restoration ecologists look further at adaptation within the zones.

## Conclusion

Serving and balancing the different interests and needs of many stakeholders and end‐users during the planning of a restoration project can be challenging. The project should be feasible for practitioners at the same time as it ensures establishment success of vegetation, often within the framework of laws and regulations. Furthermore, restoration targets may vary from ecosystems to vegetation and single species. In this study, we developed an easy and flexible system that may serve as an example on how to meet the different demands for choice of seed material for restoration of vegetation, which may well be adopted also in other geographical regions and ecosystems. To our knowledge, this is the first study to combine this many species covering a large geographic area using a gene flow approach to seed transfer zone construction. Studies published so far focus on single species restoration (e.g. Gao et al. 2012; Gibbs et al. 2012; Michalski and Durka 2012) or on regional scale (e.g. Krauss and Koch 2004; Krauss and He 2006; Malaval et al. 2010). Our study shows that dense and nation‐wide sampling of several species commonly used in restoration of vegetation in combination with highly variable and neutral genetic markers is a useful and practical approach for defining local seed provenance. The results are intended to be of immediate use to help practitioners and managers select appropriate seeds for restoration projects in compliance with the Norwegian Nature Diversity Act. For the six species in the study, four seed transfer zones suffice for Norway, which is precautionary as no species had more than two genetic groups. The species are all alpine with large amounts of gene flow; thus, we should be careful if we extrapolate from these results to lowland species or to species that are not wind‐pollinated or wind‐dispersed. Even so, for the purpose of re‐vegetation in alpine regions in Norway, our six species is quite enough. In most cases of re‐vegetation, we primarily need to establish a cover for aesthetic reasons, to avoid erosion or prevent invasion of non‐native species.

## Data archiving statement

Data available from the Dryad Digital Repository: http://dx.doi.org/10.5061/dryad.7q10s.

## Supporting information


**Table S1.** Sampling details for each locality included in this study.
**Figure S1.** Structure analyses summary.
**Figure S2.** Structure results for all species included in this study.
