# Supplementary material for: What's the meaning of local? Using molecular markers to define seed transfer zones for ecological restoration in Norway
Source: Evol Appl. 2016 Apr 6;9(5):673–84. doi: 10.1111/eva.12378 (PMC4869409; doi:10.1111/eva.12378)
Supplement: Supplementary file 1 — Table S1. Sampling details for each locality included in this study. Figure S1. Structure analyses summary. Figure S2. Structure results for all species included in this study. [file EVA-9-673-s001.docx]

# Supplementary data

**Table S1.** Sampling details for each locality included in this study.

| **Locality** | **Lat./Long. (N/E)** | **Species** | ***N*** | **Collected by** | **Date** |
| --- | --- | --- | --- | --- | --- |
| **1) Finnmark E** | 70.27/30.96 | *Agrostis mertensii* | 15 | Erling Fjelldal/Bente Sved Skottvoll | 11/07/2011 |
|  |  | *Avenella flexuosa* | 15 | Erling Fjelldal/Bente Sved Skottvoll | 11/07/2011 |
|  |  | *Carex bigelowii* | 15 | Erling Fjelldal/Bente Sved Skottvoll | 12/07/2011 |
|  |  | *Festuca ovina* | 15 | Erling Fjelldal/Bente Sved Skottvoll | 12/07/2011 |
|  |  | *Poa alpina* | 7 | Erling Fjelldal/Bente Sved Skottvoll | 12/07/2011 |
|  |  | *Scorzoneroides autumnalis* | - |  |  |
|  |  |  |  |  |  |
| **2) Finnmarksvidda** | 69.40/24.53 | *Agrostis mertensii* | 14 | Erling Fjelldal/Bente Sved Skottvoll | 13/07/2011 |
|  |  | *Avenella flexuosa* | 15 | Erling Fjelldal | 13/07/2011 |
|  |  | *Carex bigelowii* | 14 | Erling Fjelldal/Bente Sved Skottvoll | 13/07/2011 |
|  |  | *Festuca ovina* | - |  |  |
|  |  | *Poa alpina* | - |  |  |
|  |  | *Scorzoneroides autumnalis* | - |  |  |
|  |  |  |  |  |  |
| **3) Finnmark W** | 71.08/25.75 | *Agrostis mertensii* | - |  |  |
|  |  | *Avenella flexuosa* | 15 | Paul Eric Aspholm | 30/07/2011 |
|  |  | *Carex bigelowii* | 15 | Paul Eric Aspholm | 30/07/2011 |
|  |  | *Festuca ovina* | 14 | Paul Eric Aspholm | 30/07/2011 |
|  |  | *Poa alpina* | 11 | Paul Eric Aspholm | 30/07/2011 |
|  |  | *Scorzoneroides autumnalis* | 15 | Paul Eric Aspholm | 30/07/2011 |
|  |  |  |  |  |  |
| **4) Lyngen** | 69.60/20.24 | *Agrostis mertensii* | - |  |  |
|  |  | *Avenella flexuosa* | 15 | Paul Eric Aspholm | 01/08/2011 |
|  |  | *Carex bigelowii* | 14 | Paul Eric Aspholm | 01/08/2011 |
|  |  | *Festuca ovina* | - |  |  |
|  |  | *Poa alpina* | 4 | Leif Einar Støvern | 13/09/2011 |
|  |  | *Scorzoneroides autumnalis* | 15 | Paul Eric Aspholm | 01/08//2011 |
|  |  |  |  |  |  |
| **5) Lofoten/Vesterålen** | 68.34/14.65 | *Agrostis mertensii* | 15 | Andy Sortland | 07-08/09/2011 |
|  |  | *Avenella flexuosa* | 15 | Andy Sortland | 07-08/09/2011 |
|  |  | *Carex bigelowii* | - |  |  |
|  |  | *Festuca ovina* | - |  |  |
|  |  | *Poa alpina* | 7 | Andy Sortland | 07-09/09/2011 |
|  |  | *Scorzoneroides autumnalis* | 15 | Andy Sortland | 07-08/09/2011 |
|  |  |  |  |  |  |
| **6) Ofoten/Bjørnefjell** | 68.45/18.10 | *Agrostis mertensii* | 15 | Andy Sortland | 11-12/08/2011 |
|  |  | *Avenella flexuosa* | 15 | Andy Sortland | 11-12/08/2011 |
|  |  | *Carex bigelowii* | 11 | Andy Sortland | 11-12/08/2011 |
|  |  | *Festuca ovina* | 14 | Andy Sortland | 11-12/08/2011 |
|  |  | *Poa alpina* | 13 | Andy Sortland | 11-12/08/2011 |
|  |  | *Scorzoneroides autumnalis* | 15 | Andy Sortland | 11-12/08/2011 |
|  |  |  |  |  |  |
| **7) Saltfjellet** | 67.07/16.05 | *Agrostis mertensii* | 15 | Trond Skoglund | 15/08/2011 |
|  |  | *Avenella flexuosa* | 15 | Trond Skoglund | 15/08/2011 |
|  |  | *Carex bigelowii* | 12 | Trond Skoglund | 15/08/2011 |
|  |  | *Festuca ovina* | 15 | Trond Skoglund | 15/08/2011 |
|  |  | *Poa alpina* | 14 | Trond Skoglund | 15/08/2011 |
|  |  | *Scorzoneroides autumnalis* | 5 | Trond Skoglund | 15/08/2011 |
|  |  |  |  |  |  |
| **8) Børgefjell** | 65.18/13.46 | *Agrostis mertensii* | 14 | Øyvind H Opedal | 15/09/2011 |
|  |  | *Avenella flexuosa* | 15 | Øyvind H Opedal | 15/09/2011 |
|  |  | *Carex bigelowii* | 12 | Øyvind H Opedal | 14/09/2011 |
|  |  | *Festuca ovina* | - |  |  |
|  |  | *Poa alpina* | - |  |  |
|  |  | *Scorzoneroides autumnalis* | 14 | Ø H Opedal | 15/09/2011 |
|  |  |  |  |  |  |
| **9) Meråker** | 63.36/11.74 | *Agrostis mertensii* | - |  |  |
|  |  | *Avenella flexuosa* | 15 | Tor-Ivar Hansen/Dagmar Hagen | 25/09/2011 |
|  |  | *Carex bigelowii* | 14 | Tor-Ivar Hansen/Dagmar Hagen | 25/09/2011 |
|  |  | *Festuca ovina* | 14 | Tor-Ivar Hansen/Dagmar Hagen | 25/09/2011 |
|  |  | *Poa alpina* | 9 | Tor-Ivar Hansen/Dagmar Hagen | 25/09/2011 |
|  |  | *Scorzoneroides autumnalis* | 15 | Tor-Ivar Hansen/Dagmar Hagen | 25/09/2011 |
|  |  |  |  |  |  |
| **10) Kvikne/Tynset** | 62.57/10.45 | *Agrostis mertensii* | - |  |  |
|  |  | *Avenella flexuosa* | 15 | Tor-Ivar Hansen | 15/09/2011 |
|  |  | *Carex bigelowii* | 15 | Tor-Ivar Hansen | 15/09/2011 |
|  |  | *Festuca ovina* | 14 | Tor-Ivar Hansen | 15/09/2011 |
|  |  | *Poa alpina* | 6 | Tor-Ivar Hansen | 15/09/2011 |
|  |  | *Scorzoneroides autumnalis* | 15 | Tor-Ivar Hansen | 15/09/2011 |
|  |  |  |  |  |  |
| **11) Trollheimen** | 62.71/9.55 | *Agrostis mertensii* | - |  |  |
|  |  | *Avenella flexuosa* | 15 | Dagmar Hagen | 12/08/2011 |
|  |  | *Carex bigelowii* | 14 | Dagmar Hagen | 12/08/2011 |
|  |  | *Festuca ovina* | 15 | Dagmar Hagen | 12/08/2011 |
|  |  | *Poa alpina* | 13 | Dagmar Hagen | 12/08/2011 |
|  |  | *Scorzoneroides autumnalis* | 13 | Dagmar Hagen | 12/08/2011 |
|  |  |  |  |  |  |
| **12) Dovrefjell** | 62.30/9.60 | *Agrostis mertensii* | - |  |  |
|  |  | *Avenella flexuosa* | 15 | Dagmar Hagen | 14/07/2011 |
|  |  | *Carex bigelowii* | 14 | Knut Rydgren/Dagmar Hagen | 10/08/2011 |
|  |  | *Festuca ovina* | 13 | Dagmar Hagen | 14/07/2011 |
|  |  | *Poa alpina* | 15 | Dagmar Hagen | 14/07/2011 |
|  |  | *Scorzoneroides autumnalis* | 15 | Dagmar Hagen | 16/07/2011 |
|  |  |  |  |  |  |
| **13) Strynefjellet** | 62.02/7.40 | *Agrostis mertensii* | 15 | Odd Vevle | 30/07/2011 |
|  |  | *Avenella flexuosa* | 15 | Odd Vevle | 30/07/2011 |
|  |  | *Carex bigelowii* | 14 | Odd Vevle | 30/07/2011 |
|  |  | *Festuca ovina* | - |  |  |
|  |  | *Poa alpina* | - |  |  |
|  |  | *Scorzoneroides autumnalis* | 14 | Odd Vevle | 30/07/2011 |
|  |  |  |  |  |  |
| **14) Vikafjellet** | 60.93/6.43 | *Agrostis mertensii* | 15 | Odd Vevle | 27/07/2011 |
|  |  | *Avenella flexuosa* | 15 | Odd Vevle | 28/07/2011 |
|  |  | *Carex bigelowii* | 15 | Odd Vevle | 28/07/2011 |
|  |  | *Festuca ovina* | - |  |  |
|  |  | *Poa alpina* | 13 | Odd Vevle | 29/07/2011 |
|  |  | *Scorzoneroides autumnalis* | 11 | Odd Vevle | 28/07/2011 |
|  |  |  |  |  |  |
| **15) Valdresflya** | 61.34/8.81 | *Agrostis mertensii* | 15 | Odd Vevle | 31/07/2011 |
|  |  | *Avenella flexuosa* | 15 | Odd Vevle | 31/07/2011 |
|  |  | *Carex bigelowii* | 10 | Odd Vevle | 31/07/2011 |
|  |  | *Festuca ovina* | 14 | Odd Vevle | 01/05/2009 |
|  |  | *Poa alpina* | - |  |  |
|  |  | *Scorzoneroides autumnalis* | 15 | Odd Vevle | 01/08/2011 |
|  |  |  |  |  |  |
| **16) Ringebufjellet** | 61.58/10.36 | *Agrostis mertensii* | - |  |  |
|  |  | *Avenella flexuosa* | 15 | Tor-Ivar Hansen | 16/09/2011 |
|  |  | *Carex bigelowii* | 15 | Tor-Ivar Hansen | 16/09/2011 |
|  |  | *Festuca ovina* | 15 | Tor-Ivar Hansen | 16/09/2011 |
|  |  | *Poa alpina* | 10 | Tor-Ivar Hansen | 16/09/2011 |
|  |  | *Scorzoneroides autumnalis* | 15 | Tor-Ivar Hansen | 16/09/2011 |
|  |  |  |  |  |  |
| **17) Hardangervidda W** | 60.43/7.41 | *Agrostis mertensii* | 15 | Geir Flatebø | 10/08/2011 |
|  |  | *Avenella flexuosa* | 15 | Geir Flatebø | 06/08/2011 |
|  |  | *Carex bigelowii* | - |  |  |
|  |  | *Festuca ovina* | 14 | Geir Flatebø | 07/08/2011 |
|  |  | *Poa alpina* | - |  |  |
|  |  | *Scorzoneroides autumnalis* | 15 | Geir Flatebø | 06/08/2011 |
|  |  |  |  |  |  |
| **18) Hardangervidda E** | 60.24/8.53 | *Agrostis mertensii* | 15 | Odd Vevle | 05/08/2011 |
|  |  | *Avenella flexuosa* | 15 | Odd Vevle | 05/08/2011 |
|  |  | *Carex bigelowii* | 14 | Odd Vevle | 22/07/2011 |
|  |  | *Festuca ovina* | 14 | Odd Vevle | 04/08/2011 |
|  |  | *Poa alpina* | - |  |  |
|  |  | *Scorzoneroides autumnalis* | 15 | Odd Vevle | 05/08/2011 |
|  |  |  |  |  |  |
| **19) Norefjell** | 60.34/9.19 | *Agrostis mertensii* | 15 | Odd Vevle | 01/08/2011 |
|  |  | *Avenella flexuosa* | 15 | Odd Vevle | 22/07/2011 |
|  |  | *Carex bigelowii* | 13 | Odd Vevle | 01/08/2011 |
|  |  | *Festuca ovina* | 14 | Odd Vevle | 01/08/2011 |
|  |  | *Poa alpina* | 15 | Odd Vevle | 02/08/2011 |
|  |  | *Scorzoneroides autumnalis* | 15 | Odd Vevle | 02/08/2011 |
|  |  |  |  |  |  |
| **20) Setesdal/Vesthei** | 59.46/7.19 | *Agrostis mertensii* | 13 | Odd Vevle | 15/08/2011 |
|  |  | *Avenella flexuosa* | 15 | Odd Vevle | 15/08/2011 |
|  |  | *Carex bigelowii* | 8 | Odd Vevle | 15/08/2011 |
|  |  | *Festuca ovina* | - |  |  |
|  |  | *Poa alpina* | 14 | Odd Vevle | 15/08/2011 |
|  |  | *Scorzoneroides autumnalis* | 3 | Odd Vevle | 16/08/2011 |

**Figure S1.** Structure analyses summary. The figure summarizes the values for 10 independant runs for each of K=1-9 for each species. To the left: the log likelihoods. In the middle: similarity coefficiants. To the right: delta K values.

**
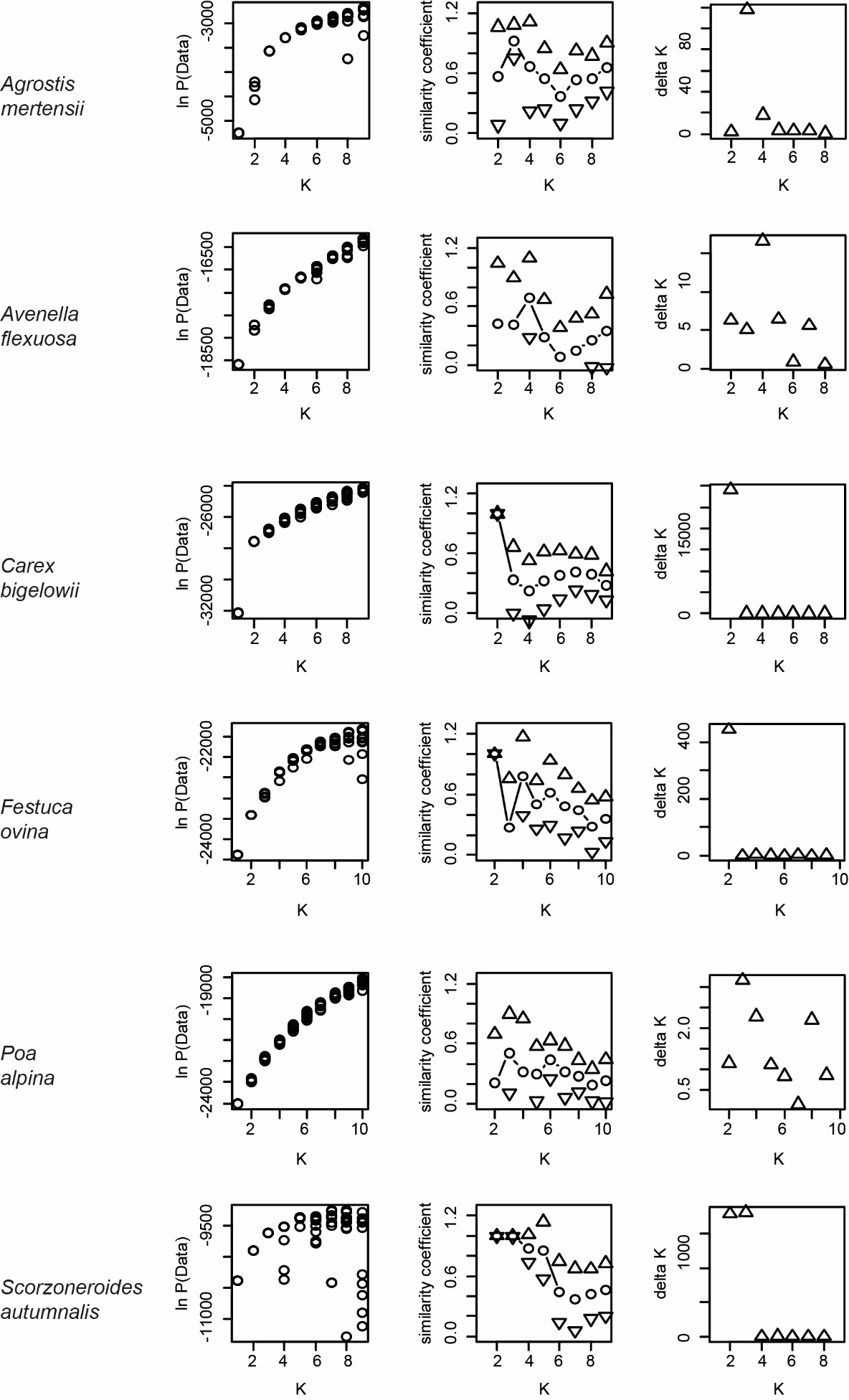
**

**Figure S2.** Structure results for all species included in this study. The plants are sorted according to population number with population 1 to the left and 20 to the right.

**
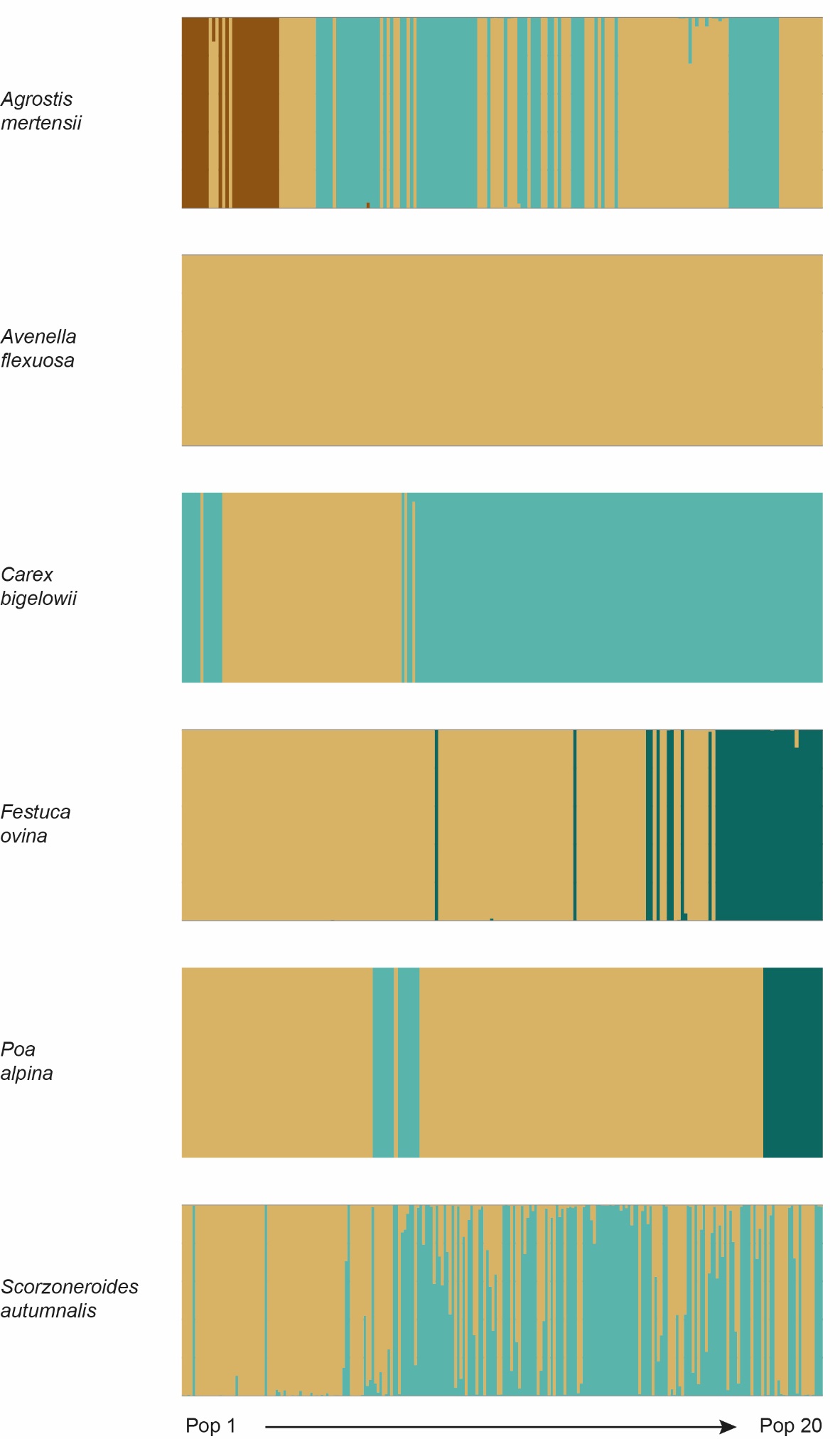
**
